# Regulatory influences of methyl jasmonate and calcium chloride on chilling injury of banana fruit during cold storage and ripening

**DOI:** 10.1002/fsn3.2058

**Published:** 2020-12-20

**Authors:** Mostafa M. Elbagoury, Losenge Turoop, Steven Runo, Daniel N. Sila

**Affiliations:** ^1^ Department of Molecular Biology and Biotechnology Pan African University Institute of Science Technology and Innovation Nairobi Kenya; ^2^ Department of Horticulture and food security Jomo Kenyatta University of Agriculture and Technology Nairobi Kenya; ^3^ Department of Biochemistry and Biotechnology Kenyatta University Nairobi Kenya; ^4^ Department of Food Science and Technology Jomo Kenyatta University of Agriculture and Technology Nairobi Kenya

**Keywords:** antioxidant activity, chilling injury, firmness, phenolic compounds, storage temperatures

## Abstract

Fruit quality is preserved through cold storage, but climacteric fruits are prone to chilling injury (CI) which limits their shelf life and marketability. Two postharvest treatments, 1 mM methyl jasmonate (MeJA) and 4% (wt/vol) calcium chloride (Ca^2+^), were separately used to investigate their influences on chilling injury (CI) incidence and fruit quality in unpacked banana cultivar “Grand Nain” during cold storage and subsequent ripening. Banana fruits were dipped for 2 min in aqueous emulsions containing 1% Tween‐80—used here as a surfactant with untreated fruits being used as control. Fruits were stored at 10 ± 2 or optimal 14 ± 2°C temperature and relative humidity 85%–90% for a 20‐day cold storage period and then removed from cold storage at 5, 10, 15, and 20 days followed by ripening at 22 ± 2°C. Treatments with MeJA or Ca^2+^ significantly reduced CI in banana fruit during cold storage and subsequent ripening temperature. Untreated controls exhibited increased CI, weight loss, and decreased hue angle, as well as firmness. In contrast, the aforementioned changes were considerably delayed after treatments with MeJA or Ca^2+^. Application of MeJA or Ca^2+^ also increased total phenolic compound contents and maintenance of total antioxidant activity throughout cold storage and during ripening periods as compared to that of the control. These findings indicate that coating bananas with 1 mM MeJA or 4% (wt/vol) Ca^2+^ can improve the postharvest quality and shelf life of fruits, and it can ameliorate chilling injury during cold storage and at ripening temperature.

## Graphical abstract

Coating the bananas with 1 mM methyl jasmonate or 4% (wt/vol) calcium chloride has the potential of improving the postharvest quality and shelf life of fruits, and it can ameliorate chilling injury during cold storage and at ripening temperature.

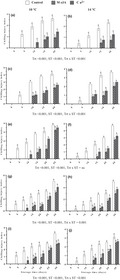

## 1 INTRODUCTION

Banana (*Musa* spp.) is a fruit of economic importance worldwide. It originated in tropical areas of the world, and its global production averaged 115 million tons in 2018 (FAO, 2018). Banana production is hindered by postharvest diseases both biotic and abiotic. Banana fruits, especially fully ripe ones, are sensitive to physical injuries during storage and transportation (Malmiri et al., 2011). Cold storage is used in various fruits and vegetables to extend their postharvest shelf life. However, many tropical and subtropical fruits are highly sensitive to chilling injury (CI) including banana. The severity of the CI damage depends on the sensitivity of packaging and unpacked banana cultivar and exposure time when stored below 10°C and near‐optimal 14–15°C (Crismas et al., 2018; Qiu et al., 2015; Zsom et al., 2018). Overall, chilling injury affects the marketability and quality of many tropical and subtropical fruits and vegetables (Cao et al., 2009).

Chilling injury is a physiological disorder, which usually enhances postharvest losses worldwide (Chen et al., 2019). Cell membrane dysfunction occurs during storage at low temperatures and finally leads to CI development in various fruits and vegetables (Rui et al., 2010), whereby cell membrane is damaged by increasing reactive oxygen species (ROS). Tolerance to CI may occur due to an increased antioxidant system that inhibits excessive ROS accumulation (Luo et al., 2015). This system includes total phenolic compounds, antioxidant activities, and antioxidant enzymes (Hosseini et al., 2018; Jiao et al., 2018).

A sharp increase in ethylene production occurs at certain stages of banana ripening. However, in commercial production, ripening is induced either by the use of commercial exogenous ethylene ripening agents or by storing in conditions where the evolution of endogenous ethylene occurs naturally (Marriott & Palmer, 1980). Changes in banana peel colors from green to yellow are obtained by slow ripening at 22°C (Wang et al., 2006). Most of the previous studies focused on the CI on bananas at the mature green stage (Jiao et al., 2018; Wu et al., 2014), and only a few reports are available on the influences of postharvest treatments on CI on green mature banana throughout cold storage and subsequent ripening.

Methyl jasmonate (MeJA) naturally occurs in a broad range of higher plants and regulates several physiological processes including the accumulation of pigments, phenolic compounds, fruit ripening, sugars, and antioxidants (Reyes‐Díaz et al., 2016; Rudell et al., 2002). Additionally, MeJA regulates various aspects of growth and plant development including fruit senescence, flowering, and ripening (Creelman & Mullet, 1995). It plays an important role in modulating plant defense response and antioxidant systems; in addition to inducing resistance against chilling injuries, it enhances secondary metabolites and antioxidant activity (Reyes‐Díaz et al., 2016). These physiological functions of MeJA have been exploited in ameliorating CI of fruits during cold storage. For example, Cao et al. (2009) reported that loquat fruit treated with 10 μmole L^−1^ MeJA reduced CI symptoms during cold storage at 1°C for 35 days was related to reservation of a high level of unsaturated/saturated fatty acid ratios and reduced lipoxygenase (LOX) activity. Furthermore, Ghiasi and Razavi (2013) found that the mitigated chilling injury is associated with the enhanced level of phenylalanine ammonia–lyase (PAL) in tomato fruits during cold storage. In addition, the application of MeJA during cold storage inhibited the development of CI symptoms in other climacteric fruits such as mandarin, peach, orange, and lemon (Baswal et al., 2020; Chen et al., 2019; Rehman et al., 2018; Siboza et al., 2014).

Similarly, postharvest application of calcium (Ca^2+^) is known to enhance tissue membrane integrity, firmness, and cell turgor which extends the storage life of fresh fruits and vegetables. Calcium is known to reduce physiological disorders and delays membrane lipid catabolism (García et al., 1996; Picchioni et al., 1998). Application of Ca^2+^ is reported to reduce and mitigate CI symptoms during cold storage through various fruit species such as loquat fruit (Li et al., 2020), banana (Jiao et al., 2018), and pomegranate (Ramezanian et al., 2010). Previously, Ca^2+^ was reported to delay browning and improve the increases in fruit firmness and pitting resistance during storage in sweet cherry (Wang et al., 2014) and apple fruit (Luo et al., 2011).

This study sought to investigate the effect of postharvest treatments with methyl jasmonate (1 mM) and calcium chloride (4% wt/vol) on chilling injury of unpacked banana at the mature green stage during low‐temperature storage 10 ± 2 or optimal 14 ± 2°C, and after transferring to ripening temperature at 22 ± 2°C. The transfer was staggered starting from the 5th, 10th, 15th, and 20th days of cold storage. The study also aimed at evaluating the occurrence of CI and the accumulation of phenolic compounds and antioxidant activity.

## 2 MATERIALS AND METHODS

### 2.1 Fruit materials

Banana fruits (*Musa* spp., AAA group cv. “Grand Nain”) were obtained from a commercial orchard at their commercial maturity (green) stage with firmness 30.0744 N. Fruits after harvest were then transferred immediately to Food Science Laboratories at Jomo Kenyatta University of Agriculture and Technology, in Kenya. Fruits selected were of high quality, healthy, had no surface contamination, and free from any visible disease symptoms or physical damage. Fruits used for the experiments were selected for uniformity in shape, size, color, and ripening stage.

### 2.2 Treatments

Selected banana fruits (variety “Grand Nain”) were divided into six groups and prepared in small uniform hands (about 4–5 fingers each). A completely randomized experimental design with three replicates (ten hands each) was established. Fruit fingers were washed with a solution of sodium hypochlorite (0.01%) for 3 min and air‐dried at room temperature (25°C). Data on banana quality and chemical analysis were collected at day zero in three samples (4 fingers of each) that were randomly collected.

Treatments for bananas were carried out as follows: Methyl jasmonate (1 mM) (≥9.5% purity, CAS number 39924‐52‐2; Sigma‐Aldrich, USA) and calcium chloride (4% wt/vol) solutions were prepared by mixing these compounds with an aqueous solution containing 0.1% (vol/vol) Tween‐80 (CAS No. 9005‐65‐6) as an emulsifier. Subsequently, banana fruits were dipped into each concentration of postharvest treatment solutions for 2 min. Control fruits were treated with a solution consisting of the 0.1% Tween‐80 aqueous solution alone (Tween‐treated). Treated fruits were stored unpacked at 10 ± 2°C or optimal 14 ± 2°C cold storage temperature and 85%–90% relative humidity (RH) for 20 days.

Chilling injury index, weight loss, firmness, Hue angle (H°), total phenol content (TPC), and total antioxidant activity (TAA) were determined. All the assessments were carried out after the start of the experiment on days 0, 5, 10, 15, and 20 during cold storage at 10 ± 2 and 14 ± 2°C. This was followed by transferring fruits from 5th, 10th, 15th, and 20th days throughout cold storage to subsequently ripening naturally at 22 ± 2°C (Wang et al., 2006). The samples were taken during cold storage and ripening condition. The peels of bananas were cut into pieces, quickly frozen in liquid nitrogen, and kept at −80°C for future use.

### 2.3 Chilling injury index measurements (CI index)

Chilling injury was analyzed by determining the extent of the browning area of fruit peel according to the following scale: 1 = no damage; 2 = very light damage; 3 = moderate damage (25% surface affected); 4 = severe damage (26%–50% surface affected); and 5 = very severe damage (>50% surface affected) as described (Lo'ay, 2005). This was then used to determine the chilling injury index using the following formula:

$$\text{Chilling Injury Index}=\sum_{1=5}^{5}\frac{\text{chilling injury level*number of fruits at the level}}{\text{total number of fruits}}$$

### 2.4 Changes in peel firmness

To determine firmness, a penetrometer (Model CR‐100D; Sun Scientific Co. Ltd, Japan) was used with unpeeled fruits. The three samples of unpeeled banana fruits from each treatment had their peel firmness measured. The mean pressure (as N) was recorded at three different spots in the fruits, in the middle, proximal, and distal parts. Peel firmness was measured using a penetrometer fitted with a 5‐mm probe. The probe was allowed to penetrate the peel to a depth of 6 mm, and the corresponding force required to penetrate this depth was determined. Firmness was then expressed as newton (N) according to (Jiang et al., 1999).

### 2.5 Changes in peel color

Peel colors were measured for three randomly sampled fruits from the different storage conditions using a Minolta color meter (Model CR‐200, Osaka, Japan) which was calibrated with a white and black standard tile. Color coordinates were obtained, that is L*, a*, and b*, and then, the hue angle (h°) was calculated by converting the a* and b*according to McLellan et al. (1995) as shown below:

$$\begin{array}{cc} {\text{Hue angle (H}}^{\circ })=\text{arctan (b/a) (for}\;+\text{a and}\;+\text{b values)}\\ & =\text{arctan (b/a)}+180\;(\text{for}\;‐\text{a and}\;+\text{b values})\\ & =\text{arctan (b/a)}+180\;(\text{for}\;‐\text{a and}\;‐\text{b values}) \end{array}$$

### 2.6 Weight loss

Banana fruits were weighed with a digital scale (0.001 g precision) at the beginning of the experiment (day 0) and 5, 10, 15, and 20 days during cold storage at 10 ± 2 and 14 ± 2°C.

Thereafter, the weight loss was also measured throughout ripening condition in transferred fruits from 5th, 10th, 15th, and 20th days during cold storage to 22 ± 2°C in each treatment. The percentage of cumulative weight loss was calculated as (weight−initial weight)/(initial weight) × 100.

### 2.7 Total antioxidant activity and total phenolic content

Tissues were taken from different parts of banana fruit peel, frozen in liquid N_2_, and 5 g of tissues was homogenized in 10 ml of phosphate buffer 50 mmol/L at pH 7.8. Then, the homogenate was centrifuged at 15,000 × *g* at 4°C for 20 min and the supernatant (fruit extract) was used for the analysis of total antioxidant activity and total phenolic content.

Total antioxidant activity was measured using free radical 2,2‐diphenyl‐1‐picrylhydrazyl (DPPH) scavenging activity assay according to Dokhanieh et al. (2013) with modification reported by (Wang & Gao, 2013). Fifty μl of fruit extract was added to 1.0 ml of 60 μmol/L DPPH (free radical, ≥95% purity; CAS number 1898‐66‐4; Sigma‐Aldrich, Germany) in methanol. The mixture was shaken and kept at room temperature in dark for 60 min, and then, absorbance was measured at 515 nm with a UV‐Visible Spectrophotometer (UV/VIS Spectrophotometer, JENWAY Model 6,800; QA, UK). Methanol was used as a control. The percent of reduction in DPPH was calculated according to the following equation, where (Abs control) is the absorbance of DPPH solution without fruit extracts.

$$\text{\textbackslash{}\% inhibition of DPPH}∙=\frac{\text{Abs control}‐\text{Abs sample}}{\text{Abs control}}\times 100$$

Total phenolic content analysis was assessed according to Mirdehghan and Rahimi (2016); 100 μl of fruit extract was mixed with 400 μl phosphate buffer 50 mmol/L at pH 7.8 and 2.5 ml of Folin–Ciocalteu reagent. After 1 min, 2 ml of Na_2_CO_3_ (7.5%) was added to the mixture and the sample kept at 50°C for 5 min, before measuring the absorbance at 760 nm with a UV‐VIS Spectrophotometer (JENWAY Model 6,800). Tannic acid was used as a standard, and results were expressed as mg of tannic acid per 100 g of fresh weight (F.W).

### 2.8 Statistical analysis

Data experiments of chilling injury index, firmness, hue angle, weight loss, total phenolic content, and antioxidant activity were analyzed for the effects of the MeJA and Ca^2+^ by subjecting to two‐way analysis of variance (ANOVA) using the GLM procedure (IBM SPSS software 23) methods and GraphPad Prism 7. Whenever the treatment effects were significant, the means were compared using Tukey's (HSD) range test. All tests were performed at the 5% level of significance.

## 3 RESULTS

### 3.1 Effect of MeJA or Ca^2+^ on the CI index of banana fruits

**FIGURE 1:**
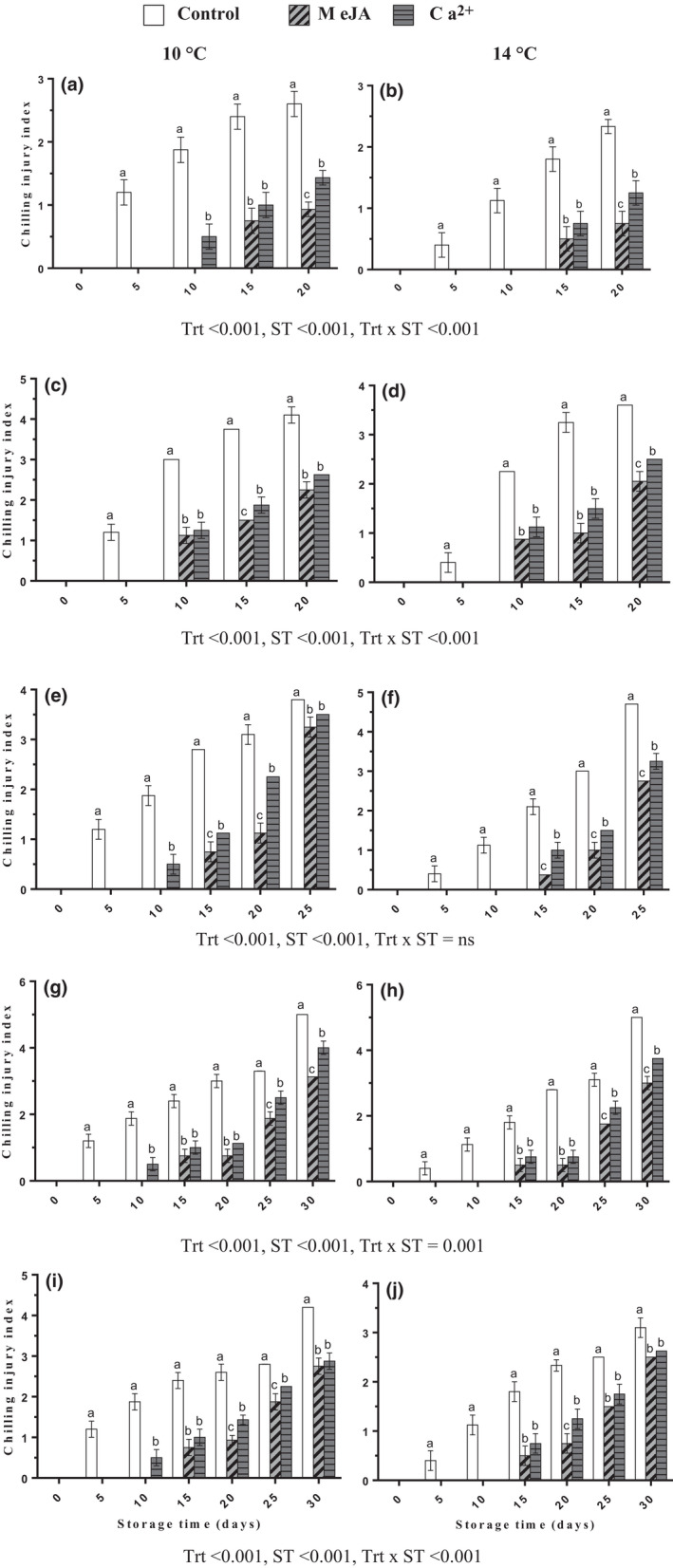
Effect of MeJA or Ca^2+^ treatment on chilling injury in bananas during cold storage (a, b) at 10 and 14°C, respectively, fruits transferred to 22°C after (c, d) 5th, (e, f) 10th, (g, h) 15th, and (i, j) 20th days of cold storage at 10 and 14°C, respectively. Statistical significance was determined at *p* ≤ .05 according to Tukey's range test (Trt = treatment, ST = storage temperature)

Chilling injury symptoms index of all bananas increased gradually during cold storage. Notably, the severity of CI in control was more rapid compared with that of treated fruit in both cold storage temperatures (10 ± 2°C and 14 ± 2°C) and natural ripening temperatures (22 ± 2°C) as shown in Figure 1. On day 20 of cold storage, dip treatments with MeJA or Ca^2+^ significantly reduced CI by 0.93 or 1.43, respectively, at 10 ± 2°C (Figure 1a) and 0.75 or 1.25, respectively, at 14 ± 2°C (Figure 1b). The CI was lower at 14 ± 2°C compared with those at 10 ± 2°C cold storage and after transferring fruits for ripening temperature. Additionally, MeJA had significantly lower CI than Ca^2+^ throughout cold storage and subsequent ripening. There was an interaction observed between treatments and storage temperatures except for transferred fruits from day 10 of both cold storage temperatures to ripening at 22 ± 2°C (Figure 1e,f). The lowest level of CI index during ripening temperature was observed in MeJA treatment in transferred fruit from 10 ± 2°C (Figure 1c,e,g and i) and 14 ± 2°C cold storage (Figure 1d,f,h and j).

### 3.2 Effect of MeJA or Ca^2+^ on peel firmness of banana fruits

**FIGURE 2:**
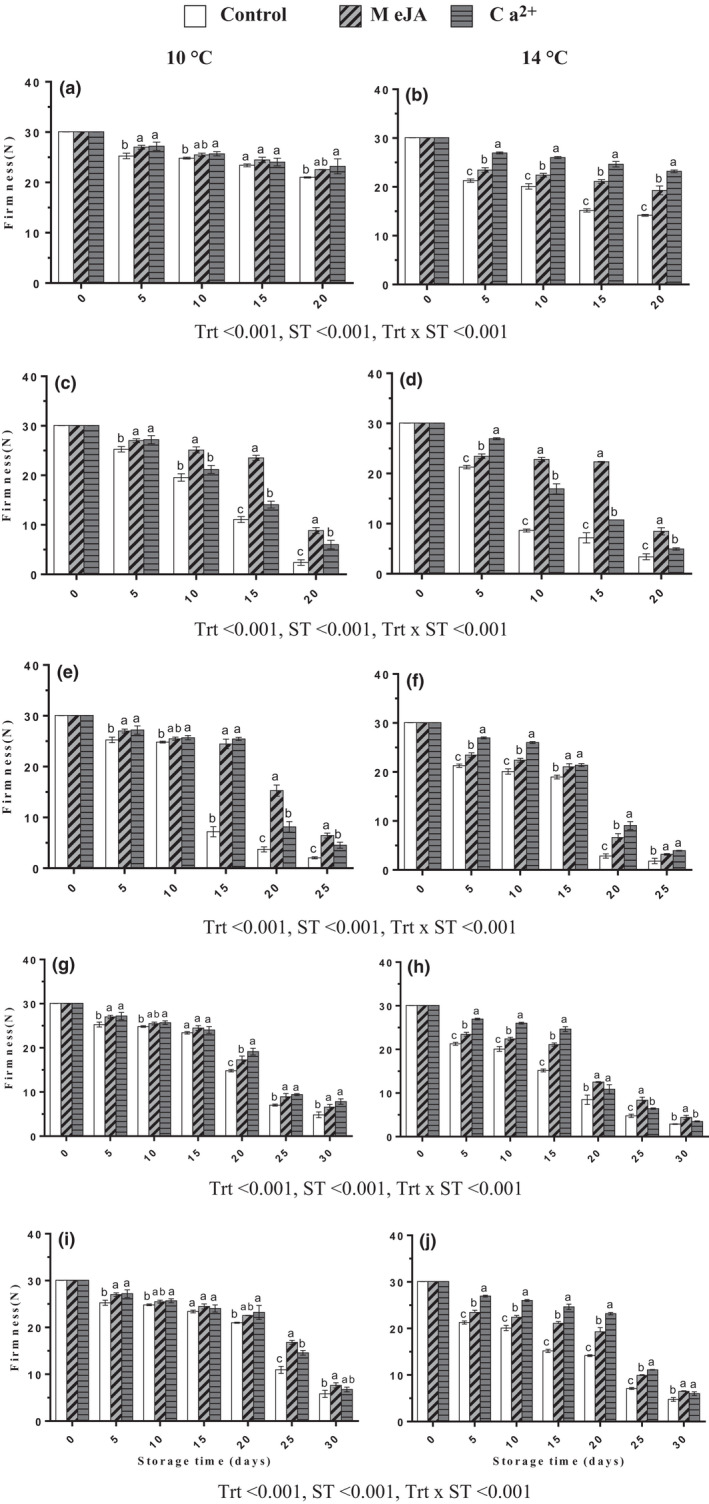
Effect of MeJA or Ca^2+^ treatment on firmness in banana peel during cold storage (a, b) at 10 and 14°C, respectively, fruits transferred to 22°C after (c, d) 5th, (e, f) 10th, (g, h) 15th, and (i, j) 20th days during cold storage at 10 and 14°C, respectively. Statistical significance was determined at *p* ≤ .05 according to Tukey's range test (Trt = treatment, ST = storage temperature)

Firmness is one of the postharvest features regarding fruit quality. Banana is a sensitive fruit that suffers a rapid loss of firmness during cold storage and ripening, and this contributes greatly to its short shelf life. Fruit firmness decreased significantly during cold storage at 10 ± 2 and 14 ± 2°C, and during ripening at 22 ± 2°C in control relative to treated samples (Figure 2). Treatment of banana fruit with MeJA or Ca^2+^ decreased the firmness to a small extent on day 20 when compared to the control by 22.55 or 23.20 N at 10 ± 2°C (Figure 2a), and by 19.29 or 23.18 N, respectively, during cold storage at 14 ± 2°C (Figure 2b). In general, peel firmness was significantly higher when the fruit was stored at 10 ± 2°C than at 14 ± 2°C, and after transferring fruits for ripening temperature. MeJA treatment led to higher fruit firmness than Ca^2+^ in transferred fruit from 10 ± 2°C cold storage to ripening temperature (Figure 2c,e and i). However, Ca^2+^ had higher firmness than MeJA in transferred fruit from cold storage at 14 ± 2°C for ripening (Figure 2f,h and j).

### 3.3 Effect of MeJA or Ca^2+^ on peel hue angle of banana fruits

**FIGURE 3:**
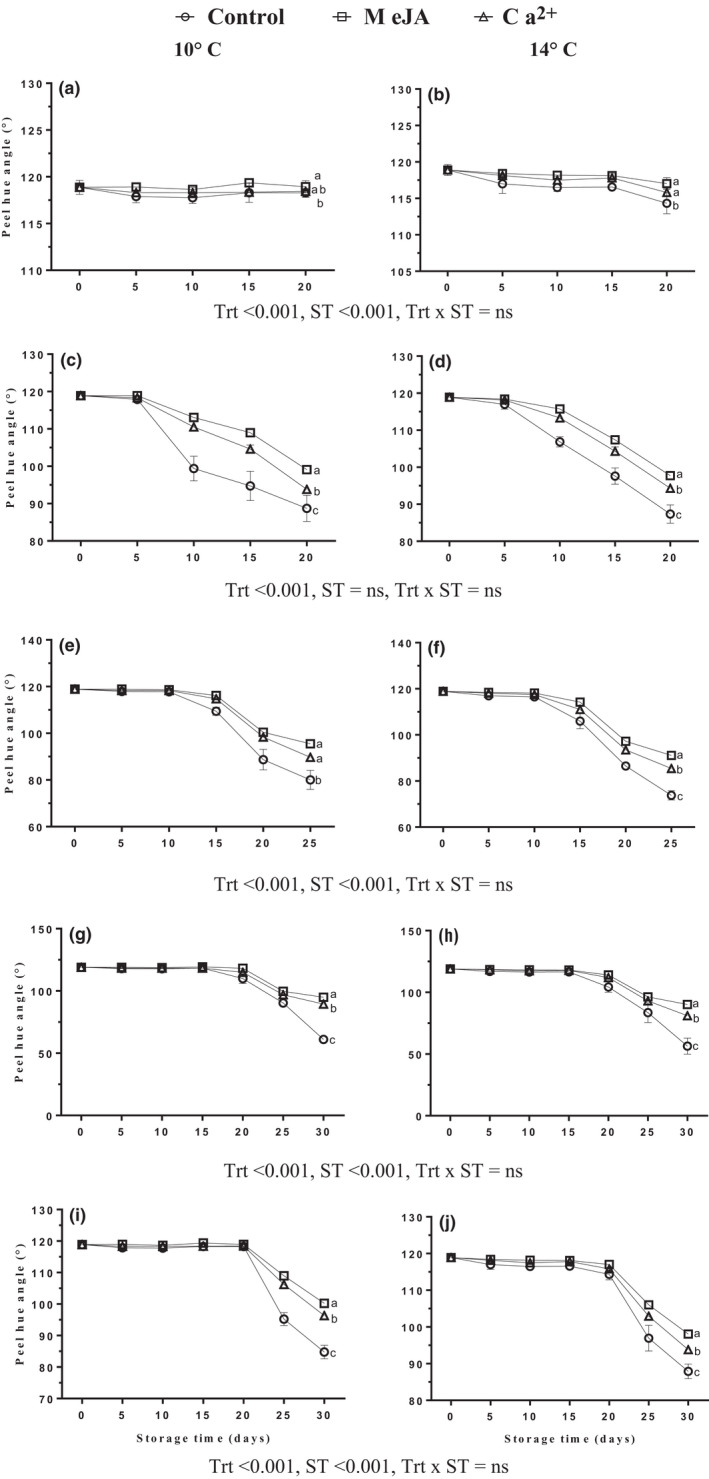
Effect of MeJA or Ca^2+^ treatment on hue angle in banana peel during cold storage (a, b) at 10 and 14°C, respectively, fruits transferred to 22°C after (c, d) 5th, (e, f) 10th, (g, h) 15th, and (i, j) 20th days during cold storage at 10 and 14°C, respectively. Statistical significance was determined at *p* ≤ .05 according to Tukey's range test (Trt = treatment, ST = storage temperature)

Peel color which is expressed as a hue angle mirrored the results of changes in the CI index. The hue angle was reduced during the periods of storage and ripening. Fruits treated with MeJA or Ca^2+^ had notably higher hue angle values compared with control fruits during cold storage at 10 ± 2°C and 14 ± 2°C, as well as after transfer to ripening temperatures at 22 ± 2°C (Figure 3). There was a notable difference observed between MeJA or Ca^2+^ treatments on hue angle values throughout cold storage and subsequent ripening. MeJA had a higher hue angle as compared to Ca^2+^‐treated samples. The average hue angle of banana treated with MeJA, Ca^2+^, or control reduced gradually from the initial value of 118.879° to 97.45°, 92.32°, or 78.65°, respectively, in transferred fruits from 10 ± 2°C of cold storage to ripening naturally (Figure 3c,e,g and i). On the other hand, it decreased to 94.25°, 88.65°, or 76.37°, respectively, in transferred fruits from cold storage at 14 ± 2°C for ripening (Figure 3d,f,h and j). Peel hue angle value was significantly higher at 10 ± 2°C compared with those at 14 ± 2°C, and after transferring fruits for ripening, there was no storage temperature effect in transferred fruits from day 5 of cold storage temperatures to ripening naturally (Figure 3c,d).

### 3.4 Effect of MeJA or Ca^2+^ on percent cumulative weight loss of banana fruits

**FIGURE 4:**
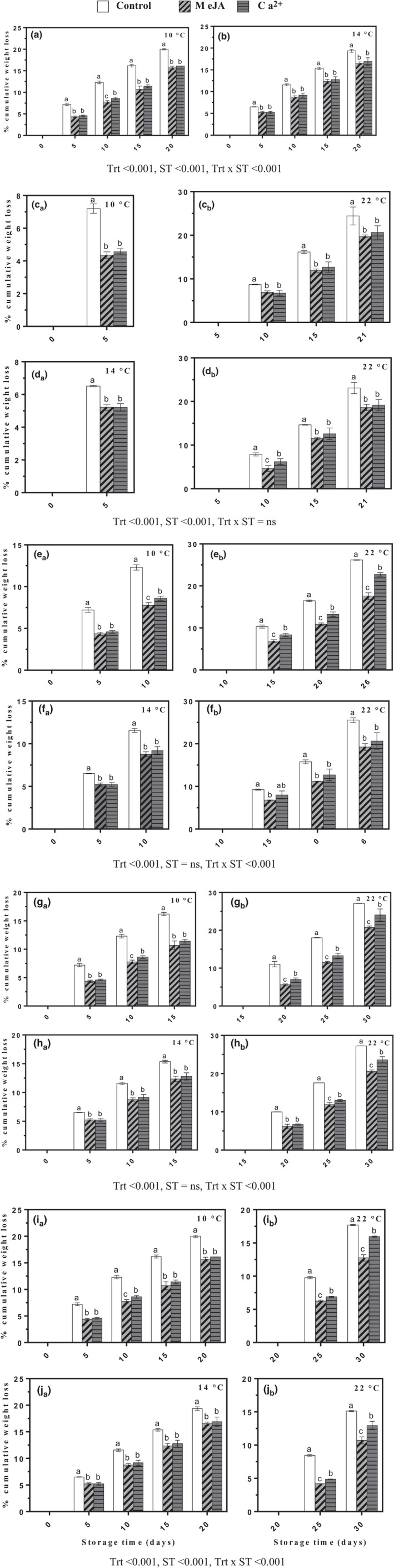
Effect of MeJA or Ca^2+^ treatment on cumulative weight loss in bananas during cold storage (a–b), (c_a_–d_a_), (e_a_–f_a_), (g_a_–h_a_), and (i_a_–j_a_) at 10 and 14°C, respectively. Transferred fruits to 22°C after (c_b_–d_b_) 5th, (e_b_–f_b_) 10th, (g_b_–h_b_) 15th, and (i_b_–j_b_) 20th days during cold storage at 10 and 14°C, respectively. Statistical significance was determined at *p* ≤ .05 according to Tukey's range test (Trt = treatment, ST = storage temperature)

Fruit transpiration is responsible for the fruit cumulative weight loss during storage. Fruits treated with MeJA or Ca^2+^ had a significantly lower percentage weight loss compared with those of the control group during cold storage and ripening periods (Figure 4). From 5‐ and 10‐ to 15‐ and 20‐day cold storage, treated fruits showed less weight loss compared with the control group. At day 20 throughout cold storage, the weight loss percentage of banana treated with MeJA, Ca^2+^, or control increased from their initial values to 15.69, 16.10, or 20.03%, respectively, at 10 ± 2°C (Figure 4a). It also increased to 16.54, 16.90, or 19.37%, respectively, at 14 ± 2°C (Figure 4b). A significant difference was observed between MeJA‐ or Ca^2+^‐treated samples during cold storage and subsequent ripening where MeJA had lower percentage weight loss as compared to Ca^2+^. The observed weight loss was higher when the fruits were stored at 14 ± 2°C compared with those that were stored at 10 ± 2°C cold storage. Moreover, the weight loss during ripening was higher in transferred fruits from 10 ± 2°C (Figure 4c_b_,e_b_,g_b_ and i_b_) than those transferred from 14 ± 2°C (Figure 4d_b_,f_b_,h_b_ and j_b_).

### 3.5 Effect of MeJA or Ca^2+^ on the total antioxidant content of banana fruits

**FIGURE 5:**
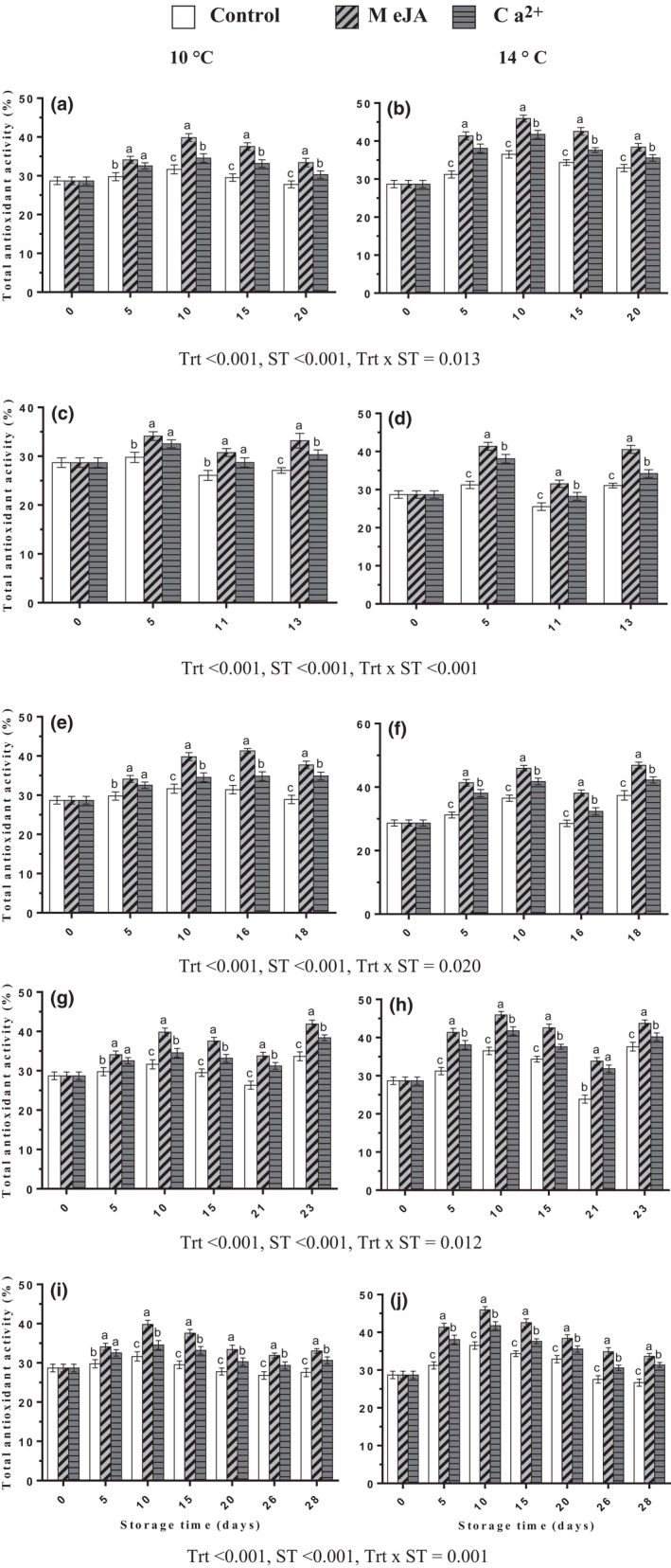
Effect of MeJA or Ca^2+^ treatment on total antioxidant activity in bananas during cold storage (a, b) at 10 and 14°C, respectively. Transferred fruits to 22°C after (c, d) 5th, (e, f) 10th, (g, h) 15th, and (i, j) 20th days during cold storage at 10 and 14°C, respectively. Statistical significance was determined at *p* ≤ .05 according to the Tukey's range test (Trt = treatment, ST = storage temperature)

The total antioxidant content is an important characteristic of the antioxidant potential during storage in banana fruits. DPPH scavenging capacity of banana fruits treated with MeJA or Ca^2+^ was significantly enhanced when compared to that of the control group during cold storage at 10 ± 2°C and 14 ± 2°C, as well as after transferring fruits to 22 ± 2°C for natural ripening (Figure 5). The results showed that treatments with MeJA or Ca^2+^ stimulated the scavenging activity of the banana fruits on DPPH free radical. The total antioxidant activity was also increased at day 5 followed by day 10, while it decreased gradually by days 15 and 20, respectively, during cold storage at 10 ± 2°C (Figure 5a) and 14 ± 2°C (Figure 5b). The total antioxidant activity was higher when the fruits were stored at 14 ± 2°C than at 10 ± 2°C and after transferring fruits for ripening. Furthermore, there was an interaction observed between treatments and storage temperatures. Treatment with MeJA had significantly higher antioxidant activity than Ca^2+^ over the cold storage and subsequently ripening. The highest antioxidant activity observed during the ripening period was 41.89% followed by 41.32% with MeJA treatment in transferred fruits from days 15 and 10, respectively, of cold storage at 10 ± 2°C for ripening (Figure 5g,e). On the other hand, it was 46.88% followed by 43.73% with MeJA in transferred fruits from days 10 and 15, respectively, at 14 ± 2°C cold storage for ripening (Figure 5f,h).

### 3.6 Effect of MeJA or Ca^2+^ on the total phenolic compound concentration of banana fruits

**FIGURE 6:**
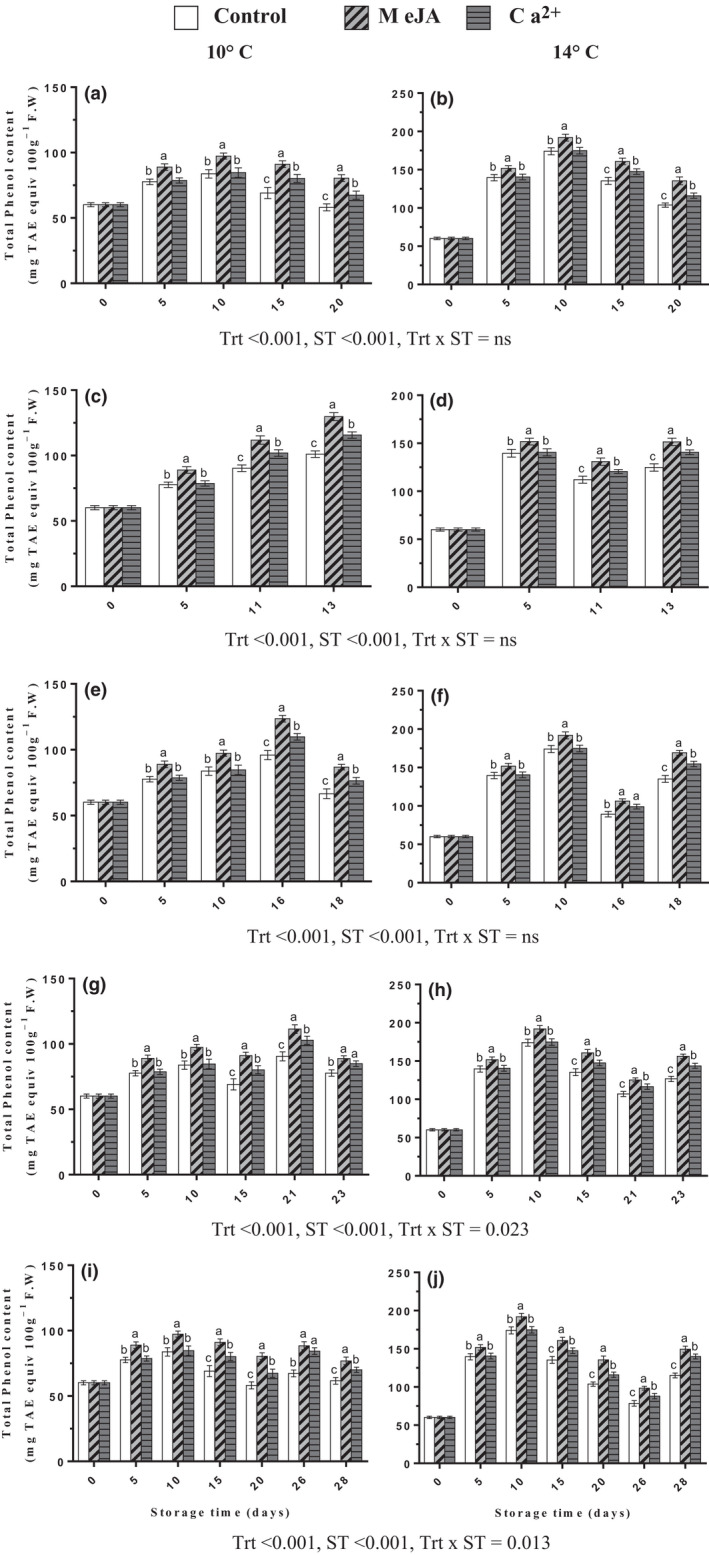
Effect of MeJA or Ca^2+^ treatment on total phenol content in bananas during cold storage (a, b) at 10 and 14°C, respectively, fruits transferred to 22°C after (c, d) 5th, (e, f) 10th, (g, h) 15th, and (i, j) 20th days during cold storage at 10 and 14°C, respectively. Statistical significance was determined at *p* ≤ .05 according to Tukey's range test (Trt = treatment, ST = storage temperature)

The concentration of total phenolic compounds is an important component of the antioxidant capacity of banana fruits. The contents of total phenols of banana fruits treated with MeJA or Ca^2+^ were significantly enhanced when compared with the control fruit during postharvest cold storage at 10 ± 2°C and 14 ± 2°C, and after transferring fruits to ripening temperature at 22 ± 2°C (Figure 6). The results showed that the total phenolic compound contents increased in all fruits by day 5 and continued to rise by day 10 followed by a decrease in days 15 and 20 during cold storage at 10 ± 2 and 14 ± 2°C (Figure 6a,b). Contents of total phenols of banana fruits treated with MeJA were higher than those treated with Ca^2+^ during cold storage and subsequent ripening. Moreover, TPC was higher when the fruit was stored at 14 ± 2°C than at 10 ± 2°C. The interaction between treatments and storage temperatures was observed during the ripening period in only transferred fruit from days 15 and 20 of cold storage to ripening naturally (Figure 6g,h,i and j). The highest phenolic compound content during the ripening period was 129.767 followed by 123.569 mg TAE equiv 100 g^‐1^ F.W with MeJA treatment in transferred fruits from days 5 and 10, respectively, of cold storage at 10 ± 2°C for ripening (Figure 6c,e). Also, it was 169.238 followed by 156.124 mg TAE equiv 100 g^−1^ F.W by MeJA in fruits transferred from days 10 and 15, respectively, at 14 ± 2°C cold storage to ripening naturally (Figure 6f,h).

## 4 DISCUSSION

The main site for CI is cell membrane followed by membrane disruption and membrane integrity losses (Li et al., 2012). Possibly, the coating of fruit samples with MeJA leads to the protection of banana fruit from membrane damage. This also may enhance the chilling tolerance of fruits through the improved activity of PAL, total antioxidant activity, and total phenolic compounds in the flavedo tissue.

MeJA treatments proved to decrease the incidence of visible CI symptoms and improve chilling tolerance in several economical fruits (Sayyari et al., 2011). In addition, MeJA application increased the endogenous content of jasmonic acid (JA) and expression levels of JA biosynthetic genes, which indicate that MeJA could reduce the increase in CI effectively during cold storage in banana fruit (Zhao et al., 2013). While treatments with Ca^2+^ were reported to be responsible for the increased calcium content in the cytoplasm and may help to improve cold stress tolerance in fruits by maintaining the plasma membrane integrity (Gang et al., 2015). Previously, Jiao et al. (2018) reported that higher TPC and a relatively lower PPO activity were observed in bananas treated with Ca^2+^ than control fruits. This treatment might have been responsible for the inhibition of peel brown discoloration and the increased antioxidant capacity, which has been linked to a reduction of CI of banana during cold storage. Increases in phenolic content and total antioxidants due to attribution to activation of PAL were involved in reducing CI index in the flavedo tissue during cold storage of lemon (Siboza et al., 2014). Additionally, alleviation of CI may be attributed to the enhancement of individual phenolic compounds during cold storage (Wang et al., 2019). Generally, an increase in PAL activity in fruit stored at chilling and low temperatures is part of plant organ response in order to alleviate CI (Ghiasi & Razavi, 2013). Rehman et al. (2018) observed that irrespective of the concentration, MeJA applications resulted in a reduced CI and an increase in the quality of sweet oranges. Mirdehghan and Ghotbi (2014) reported that alleviating chilling injury in pomegranate fruits during cold storage was a result of MeJA and Ca^2+^ treatments. Additionally, Khaliq et al. (2015) pointed out that calcium can retard the incidences of CI and an overall increase in the chilling tolerance of mango fruits. The CI symptom appearance is often accompanied by an increase in lipoxygenase (LOX) activity. This may be because LOX catalyzes peroxidation of polyunsaturated fatty acids which is believed to be the main contributor to chilling‐induced membrane damage in plant tissue (Pinhero et al., 1998). Cao et al. (2009) found that higher unsaturated/saturated fatty acid ratios and lower LOX activity were associated with a decrease in the chilling injury in MeJA‐treated loquat fruits. In general, the accumulation of excess reactive oxygen species (ROS) and the percentage of lipid peroxidation in the cell membrane are associated with a CI exacerbation in vegetables and fruit (Jiao et al., 2018). The chilling tolerance of plants may be associated with the increasing antioxidant system to prevent excessive accumulation of ROS (Luo et al., 2015). The antioxidant system, ingredients enzymatic and non‐enzymatic constituents, plays an important role in increasing chilling tolerance and scavenging ROS in many fruits (Jimenez et al., 2002). Some studies have found the appearance of CI symptoms due to the presence of ethylene in fruit storage at low temperatures. For instance, the development of CI symptoms became more evident in avocado fruit under a combination of the presence of low temperature and ethylene in the tissue (Pesis et al., 2002). The use of MeJA or Ca^2+^ in pomegranate fruit during low temperature and ripening successfully reduced CI symptoms (Mirdehghan & Ghotbi, 2014).

Firmness is one of the important features during postharvest processes regarding fruit quality, storage potential consumer acceptance, and market values (Valero et al., 2003). Possibly, the retention of higher firmness influenced by postharvest treatments with MeJA and Ca^2+^ compounds may be attributed to higher levels of antioxidants activity and total phenol contents (Figures 5 and 6), respectively. Previously, MeJA was the most effective in increasing the firmness in mandarin fruits during cold storage (Baswal et al., 2020). Besides, MeJA throughout postharvest applications maintained higher firmness in apricot fruit (Ezzat et al., 2017). MeJA treatment improved the quality of papaya by delaying ripening and preventing firmness loss (González‐Aguilar et al., 2003). However, Shalan (2020) indicated that the peach fruits treated with Ca^2+^ significantly delayed firmness losses and weight losses either at cold storage or ambient condition. Also, Jain et al. (2019) reported that Ca^2+^ application is associated with higher retention of fruit firmness due to attributed delay in cell wall hydrolysis by mediated calcium chloride contents.

Some studies showed that applications of MeJA 0.1–10 mM could induce changes in the color of fruits by enhancing carotene accumulation and degrading chlorophyll content via promoting ethylene biosynthesis (Fan et al., 1998). Furthermore, MeJA treatment consistently showed higher values of hue, chroma, and lightness reflecting less CI symptoms than control in banana fruit (Zhao et al., 2013). However, postharvest treatment with Ca^2+^ played an inhibition role in the decline of lightness in mature green and ripening banana during cold storage (Jiao et al., 2018).

Postharvest weight losses in fruit and vegetables can result from respiration processes and transpiration (Van Hung et al., 2011). Possibly, the postharvest application of MeJA and Ca^2+^ retained a higher percentage of fruit initial weight and reduced weight loss may be due to the effects of these compounds on increasing firmness, maintaining cellular integrity, and delaying ripening and senescence. Previously, postharvest treatment with MeJA decreased weight loss in mandarin fruits during cold storage (Baswal et al., 2020). Additionally, postharvest applications of MeJA in “Arrayana” mandarines have shown smaller weight losses (Gómez et al., 2017). Weight loss of fruit was reduced in sweet orange fruit during cold storage by MeJA irrespective of the concentration (Rehman et al., 2018). However, Ca^2+^ treatment was the most viable in maintaining honey peach fruit quality and retarding weight loss rate during cold and subsequent ambient temperature storage (Gang et al., 2015).

We found that a slightly higher TPC and a relatively higher TAA than control were observed in banana fruit treated with MeJA and Ca^2+^; these results may be helpful to inhibition of the brown discoloration and promotion of total antioxidants capacity in the peel, which may result in reducing the CI of banana during cold storage at 10 and 14°C, and after transferring fruits from cold storage to 22 ± 2°C for natural ripening. PPO enzyme is believed to be a major cause of the brown discoloration by phenolic substrates oxidation in banana fruits during storage (Jiao et al., 2018). Higher activity of PPO was observed at chilling temperatures in banana fruits, and it may be the main factor in the browning reaction during cold storage; PPO enzymes have been often found in the chloroplasts, where they are related to the internal thylakoid membranes (Nguyen et al., 2003). Phenolics are important antioxidant compounds, which could inhibit the overproduction of ROS (Velioglu et al., 1998). Mirdehghan and Ghotbi (2014) found that TPC and TAA were not influenced by MeJA or Ca^2+^ treatments during cold storage in pomegranate fruits. With prolonged storage time, TAA increased, probably due to the increased punicalagin and anthocyanin as the major phenolic compound that contributes to TA activity (Kulkarni et al., 2004). In this study, fruits treated with MeJA or Ca^2+^ showed an increased level of total antioxidants in a banana during cold storage and ripening as compared with the control. Similarly, Rehman et al. (2018) have shown that enhanced antioxidant activity through MeJA termites might improve the functional properties of fruit during storage. Such correlation was observed between total antioxidant activity and total phenolic content in pomegranate fruits treated with Ca^2+^ during cold storage (Ramezanian et al., 2010). Furthermore, Cao et al. (2009) found that postharvest applications with MeJA maintained higher antioxidant activity and exhibited higher levels of total phenolic in loquat fruit as compared to the control, while total phenolic content and total antioxidant activity increased in pomegranate fruits treated with Ca^2+^ during cold storage at 2°C and after held fruits at 20°C (Ramezanian et al., 2010). The mode of action of postharvest MeJA or Ca^2+^ applications in regulating total phenolic content and total antioxidant activity levels in fruit throughout cold storage temperatures is yet to be investigated.

## 5 CONCLUSIONS

In conclusion, 1 mM MeJA and 4% (wt/vol) Ca^2+^ dip applications for 2 min could alleviate CI and reduce weight loss of unpacked banana fruits throughout cold storage and after transferring for ripening naturally. MeJA was more effective to reduce chilling tolerance than Ca^2+^ during storage in both cold and ripening temperatures. According to spectrophotometry data, the influences of MeJA or Ca^2+^ on chilling tolerance are supposedly due to altering phenolic compounds and antioxidant contents in the fruit.
